# Supplementary material for: Feasibility of Combining Transcranial Direct Current Stimulation and Active Fully Embodied Virtual Reality for Visual Height Intolerance: A Double-Blind Randomized Controlled Study
Source: J Clin Med. 2022 Jan 11;11(2):345. doi: 10.3390/jcm11020345 (PMC8779186; doi:10.3390/jcm11020345)
Supplement: Supplementary file 1 [file jcm-11-00345-s001.zip › jcm-1496128-supplementary.pdf]

Supplementary Material S1: Link to view the virtual environment of our study.

https://uncloud.univ-nantes.fr/index.php/s/MZsjMzmxDzmMJwx

Supplementary Material S2: Step by step description of the implementation of virtual reality and tDCS in our study.

At the screening/inclusion visit (V1, on monday):

- Inclusion/exclusion criteria check (including the scores of the online questionnaire)
- Completion of clinical scales before VR (AQ, ATHQ, HIQ, STAI, CGI)
- Basal cortisol before VR with a spit (2 minutes in the mouth) that soaks under the tongue before the start of the VR
- Virtual task : climbing 9 levels in an elevator in a 150 meter building with a subjective discomfort rating (0 to 100) at each height level (x2 with a minimum interval of 30 seconds between SUD evaluation and after having spent 30 seconds at the corresponding level) : confirmation of a sufficiently high level of height-induced anxiety
- Basal cortisol after VR with a spit (2 minutes in the mouth) that soaks under the tongue after VR is over
- Completion of clinical scales after VR (AQ, ATHQ, HIQ, STAI, CGI)
- Explanation of procedures (VRET+tDCS/sham during V2 and V3, objectives and modality of the study)
- Signed consent

For both VRET+tDCS or VRET+sham exhibition sessions (depending on the group)

Before de session (V2 and V3 on Tuesday and Wednesday respectively) :

1. Preparation of the material
- Room configuration :
    - Set up a table with two powerful PC1 and PC2 (with VR & XSplit software)
    - Connecting the HTC headset to the PC
    - Installing the base stations
    - Turn on the joysticks
    - Perform the SteamVR calibration
    - Turn on the tracker
    - Check the side of the elevators by running the application once. This can be done without necessarily "calibrating" the board

2. Preparation with the patient
- Patient reception
- Information on the course of the session in a different room from the VRET room

b. Course of a session (1h)

- Preparation
    - Start Xsplit on PC1
    - Start the NIC software on PC2 and log in with the right parameters
    - Calibration of the virtual environment on PC1 (using VR software) with the board and the stool
    - In parallel, placement of the electrodes/tDCS cap in an airlock, the subject does not see the room with the VR cameras and the board
    - End of the calibration and placement of the electrodes
    - The patient is equipped with the Vive headset, audio headset and trackers
    - Start of the session, the subject enters the room

- VRET session
  - The patient is in the presence of a virtual environment (see supplementary material 1). He is in an urban environment with buildings. The patient is instructed to climb as high as possible.
  - At the time of the VRET launch, launching of the stimulation (active or placebo in a randomized way)
  - At the beginning, the patient gets used to the immersion for 1 min, and we check the good tolerance of the VR (the patient has no dizziness, no painful complaints, no nausea, no headaches)
  - The patient then starts the task (warned by a message on the screen). He goes up in an elevator of several floors. He has to cross a footbridge between two buildings if he wants to access an elevator on the other side and thus climb higher (about 5 meters steps) (see figure 5 and supplementary material 1)
  - At each floor, the subject visually scores the numbers corresponding to his subjective feeling of discomfort (SUD between 0 and 10) using a fixation/eye-tracking system. At each level the subject is advised to wait until the score decreases by 50% before continuing to climb. The subject will walk on a real board to accentuate the realism of the situation but without risk since it will be at ground level with the experimenter at his side (see figure 3).
  - If the patient takes a side step, equivalent to a fall, a black screen is displayed.The experimenter accompanies the patient to a corner of the room outside the elevator zones and we start again from the floor. If one presses to go up on the elevator, one returns to the floor before the fall.
  - The experimenter verifies that everything goes well. At any time the subject can call the experimenter who can manually interrupt the task (black screen) and resume it where the subject was then.
  - In parallel, the PC2 with NIC 2.0 allows the experimenter to turn off the stimulation immediately at any time (pause button).

- End of session
  - After 20 minutes, a message appears on PC1 (OR) PC2 indicating that the stimulation is over
  - Close the simulation on PC1
  - Stop XSplit on PC1
  - We remove the Vive helmet and the trackers from the patient
  - Assessment of VR tolerance (SSQ) and immersion quality (IPQ) after VRET

- End visit (V4 on Friday)
  - Completion of clinical scales before VR (AQ, ATHQ, HIQ, STAI, CGI)
  - Basal cortisol before VR with a spit (2 minutes in the mouth) that soaks under the tongue before the start of the VR
  - Virtual task : climbing 9 levels in an elevator in a 150 meter building with a subjective discomfort rating (0 to 100) at each height level (x2 with a minimum interval of 30 seconds between SUD evaluation and after having spent 30 seconds at the corresponding level)
  - Basal cortisol after VR with a spit (2 minutes in the mouth) that soaks under the tongue after VR is over
